# From operant learning to arbitrarily applicable relational responding: a review of Machine Psychology with the non-axiomatic reasoning system

**DOI:** 10.3389/frai.2026.1775040

**Published:** 2026-03-25

**Authors:** Robert Johansson, Patrick Hammer

**Affiliations:** 1 Department of Psychology, Stockholm University, Stockholm, Sweden; 2 Department of Brain and Cognitive Sciences, Massachusetts Institute of Technology, Cambridge, MA, United States

**Keywords:** arbitrarily applicable relational responding, artificial general intelligence, cognitive architecture, functional equivalence, Machine Psychology, non-axiomatic reasoning system, operant conditioning, relational frame theory

## Abstract

Machine Psychology is an emerging interdisciplinary framework that integrates principles from learning psychology with a cognitive AI architecture to advance Artificial General Intelligence (AGI) research. This article provides a focused review of Machine Psychology, tracing the progression from basic operant learning to advanced symbolic reasoning within the Non-Axiomatic Reasoning System (NARS). We first outline the theoretical foundations in operant conditioning and Relational Frame Theory, highlighting how adaptive behavior and arbitrarily applicable relational responding (AARR) serve as cornerstones of human cognition. We then describe the architecture and capabilities of NARS and its variant OpenNARS for Applications (ONA), which enable real-time sensorimotor reasoning under conditions of uncertain knowledge. Four successive experimental studies are reviewed in detail: (1) Operant conditioning tasks demonstrate that NARS can learn from reinforcing consequences to modify its behavior, achieving 100% correct responses and adapting when contingencies change. (2) In generalized identity matching, NARS abstracts an identity relation that successfully generalizes to novel stimuli after minimal training. (3) A functional equivalence study shows NARS grouping stimuli by shared consequences, such that new learning transfers spontaneously between equivalent stimuli. (4) Finally, NARS is extended to model AARR, exhibiting derived symmetric and transitive relations and context-sensitive relational reasoning (e.g. same–opposite relations) with associated transformations of stimulus functions. We discuss how specific NARS mechanisms (e.g. temporal inference, variable term introduction, relational implication) map onto psychological processes underlying learning and cognition. Machine Psychology is presented as a developmental roadmap toward human-like AGI, incrementally building cognitive skills from basic adaptation to complex symbolic reasoning. We critically evaluate the strengths and limitations of this approach and outline open research directions toward achieving flexible, theory-of-mind-capable intelligence.

## 1 Introduction

Achieving human-level cognition in artificial systems remains the grand challenge of Artificial General Intelligence (AGI) research. Traditional AI approaches often address isolated aspects of intelligence without a unifying developmental framework. In response, Machine Psychology has been proposed as an interdisciplinary paradigm that unites principles from learning psychology with cognitive architectures in AI to methodically build up general intelligence (Johansson, 2024a). This approach is motivated by the insight that human cognitive abilities emerge through continuous adaptation to the environment, and that a theory-driven roadmap of progressively complex learning capabilities could guide the creation of more human-like AGI systems (Johansson, 2020). The key idea is to start with fundamental learning processes observed in animals and humans—such as operant conditioning—and incrementally incorporate higher-order cognitive skills—such as abstract relational reasoning—into an AI system (Johansson, 2024b). By using well-established paradigms from psychology as testbeds, Machine Psychology aims to evaluate and improve an AI's ability to learn and reason in ways that parallel natural cognition (Johansson, 2019a, 2024b).

This review provides a focused account of the authors' own Machine Psychology research program, which has been developed and tested using NARS and its embodied variant ONA. It is not intended as a broad survey of AI approaches to learning or relational reasoning, but rather as a detailed examination of how one specific cognitive architecture can be progressively endowed with psychologically grounded learning capabilities.

The review focuses on the implementation of Machine Psychology within the Non-Axiomatic Reasoning System (NARS), a cognitive architecture designed for adaptive reasoning under uncertainty (Wang, 2006, 2013). NARS operates according to the Assumption of Insufficient Knowledge and Resources (AIKR), meaning it continuously learns and makes the best possible decisions with limited information and time (Wang, 1995, 2022). These properties make NARS an attractive platform for modeling psychological learning processes, since it faces constraints analogous to those of biological organisms (limited knowledge, finite memory, real-time demands). We examine how NARS (and its embodied variant OpenNARS for Applications, ONA) can be endowed with capabilities inspired by operant learning and Relational Frame Theory (RFT), thereby exhibiting increasingly complex behaviors akin to those seen in animals and humans (Johansson, 2024b). The review is aligned with the broader effort to integrate knowledge, reasoning, and even theory of mind in AI, by showing how an AI system can acquire knowledge from interaction (experience) and integrate it with a reasoning framework to produce intelligent behavior.

The article is organized as follows. Section 2 introduces operant conditioning from psychology and how adaptive behavior is shaped by consequences. Section 3 discusses Relational Frame Theory, focusing on arbitrarily applicable relational responding (AARR) as a learned basis for human symbolic cognition. Sections 4, 5 describe the Non-Axiomatic Reasoning System and its extension OpenNARS for Applications, detailing their architectures and reasoning capabilities. In Sections 6–9, we review a series of empirical studies in Machine Psychology that progressively integrate psychological principles into NARS: starting with basic operant learning (Section 6), then generalized identity matching (Section 7), functional equivalence (Section 8), and finally full AARR including derived relations and contextual control (Section 9). Section 10 provides a detailed mapping between NARS's computational mechanisms and psychological processes, illuminating how temporal inference, variable introduction, relational operators, and other NARS features correspond to learning phenomena. Section 11 discusses Machine Psychology as a developmental roadmap for AGI, drawing parallels to cognitive development and highlighting how this staged approach can produce increasingly sophisticated intelligence. Finally, Section 12 offers a critical evaluation of the framework's strengths and limitations, and suggests open research directions—including steps toward incorporating theory-of-mind capabilities and more complex real-world learning—before concluding the review.

## 2 Operant learning and adaptive behavior

In the behavioral psychology tradition, operant learning (or operant conditioning) is a fundamental process by which the behavior of organisms changes based on the consequences it produces, enabling organisms to adapt to their environments. First systematically studied by B.F. Skinner, operant conditioning is defined by the three-term contingency: an antecedent cue or discriminative stimulus (Sd), an organism's response or action (R), and a resultant stimulus (Sr) that follows the action (Skinner, 1938, 1953). When a behavior is followed by a reinforcing consequence, the probability of that behavior occurring again in the presence of the antecedent cue is increased; conversely, behaviors followed by no reinforcement or by punishing consequences decrease in frequency. In this way, behavior is “selected by its consequences,” an evolutionary analogy that highlights how operant conditioning shapes behavior adaptively over time (De Houwer and Hughes, 2020). Crucially, this form of learning does not require explicit instruction or prior knowledge—it is an experiential learning process in which the agent discovers which actions lead to beneficial outcomes in a given context.

Operant learning enables organisms to adjust to changing environments by forming functional relations between actions and outcomes. For example, a rat might learn to press a lever (R) when a light is on (Sd) if lever-pressing produces food (Sr). The rat's behavior changes through trial and error: initially the lever press may occur accidentally, but as it is followed by food, the animal becomes more likely to press the lever whenever the light appears. The same principle applies across a wide range of adaptive behaviors in animals, including humans—from a child learning to say “please” to receive a treat, to a bird learning which flowers yield nectar (Brembs, 2003). The operant paradigm emphasizes that intelligent behavior can emerge without an explicit teacher, guided only by the reinforcement structure of the environment. In essence, the environment “shapes” behavior by providing feedback.

Modern characterizations of learning often generalize these concepts beyond biological organisms. For instance, De Houwer and Hughes (2023) reconceptualize behaviors as state transitions in a system, which can include not only animals but also machines and even genetic or group systems. In their functional definition, a behavior is any observable transition in the state of a system in relation to stimuli, and learning is a change in the probability of behaviors due to regularities in experiences (De Houwer and Hughes, 2023). This broad view underscores that the same operant principles—linking actions to consequences—can apply to artificial agents. If we treat a robot or AI system as a “learning organism,” we can describe its internal state updates and actions in operant terms (state transitions reinforced or penalized by outcomes). This perspective supports the idea that operant conditioning can serve as a basis for adaptive machine behavior just as it does for animals (Johansson, 2024b). Notably, classic critiques argued that operant conditioning alone could not account for higher cognitive functions such as language acquisition (Chomsky, 1959). However, Relational Frame Theory (RFT) has since provided a behavioral account demonstrating how operant principles, extended to include relational learning, can explain these phenomena (Hayes et al., 2001, 2021). Contemporary contextual behavioral science thus treats operant conditioning as necessary groundwork upon which more sophisticated cognitive skills—including language and symbolic reasoning—can be built (Hayes et al., 2021).

In summary, operant learning provides a theoretical foundation for adaptive cognition. It demonstrates how an intelligent agent can autonomously acquire new behaviors suited to its environment through feedback, without relying on pre-programmed knowledge. For AGI research, this suggests a starting point: an artificial system should at minimum be capable of operant-like learning—dynamically updating its behavior policies based on success or failure in achieving goals (Wang, 2022). The Machine Psychology approach thus begins by ensuring the AI can learn from consequences in an online, trial-and-error fashion (Johansson, 2024b). In the next sections, we introduce Relational Frame Theory, which addresses the leap from such basic learned behaviors to the symbolic and relational complexity of human language and reasoning.

## 3 Relational frame theory and arbitrarily applicable relational responding

While operant conditioning explains how simple behaviors are learned, human intelligence exhibits abilities far beyond rote stimulus–response patterns. In particular, humans can relate stimuli in flexible, context-dependent ways that are not solely based on the physical properties of those stimuli or direct associations. Relational Frame Theory (RFT) is a modern behavioral account of human language and cognition that extends operant principles to explain this relational learning (Hayes et al., 2001). RFT posits that through a history of reinforced interactions, humans learn to respond to stimuli in terms of their relationships (sameness, difference, comparison, opposition, etc.), and crucially, these relations can be applied arbitrarily—meaning based on contextual cues rather than inherent features of the stimuli. This capability is termed Arbitrarily Applicable Relational Responding (AARR), and it is considered a hallmark of higher cognition (Hayes et al., 2001, 2021).

A classic example of AARR is language itself. The spoken word “apple” bears no intrinsic similarity to the fruit it represents, yet a person can learn to relate the sound to the object in a frame of equivalence (the word means the fruit). Once this relation is learned, a host of derived relations follow: hearing “apple” might evoke the taste or image of an apple (because the word and fruit are interchangeable in many contexts), and new functions can transfer (someone who learns that a certain apple is poisonous would avoid the fruit upon hearing “apple” even without seeing it). This goes beyond simple association; it reflects an ability to treat one stimulus as another in a given context, an ability learned and honed through experience with language and symbolic interaction (Hayes et al., 2001). According to RFT, such relational behaviors are themselves learned operants—that is, we learn not only to respond to stimuli but also to respond to relationships between them (Hayes et al., 2021). For example, a child might be reinforced for correctly identifying that “A is the same as B” in a training context, thereby learning a frame of coordination (equivalence). Over time, the child can apply this sameness relation arbitrarily to new pairs of stimuli (e.g., recognizing that if A = B and B = C, then A = C, even if A, B, C are novel symbols). Each distinct type of relation (sameness, opposition, comparison, hierarchy, etc.) is called a relational frame, and RFT catalogs many such frames that humans can learn.

Key phenomena of relational responding include mutual entailment (bidirectionality) and combinatorial entailment (transitivity and more complex inferences). Mutual entailment means that if a relation is trained in one direction (e.g., A is related to B in a certain way), a reciprocal relation can be derived (B related to A). Combinatorial entailment means that given two or more trained relations that share a common element (A → B and B → C), a new relation between the non-common elements can be inferred (A → C). Another important outcome is transformation of stimulus functions: if one stimulus in a relational network acquires a certain meaning or function (e.g., Stimulus B is associated with a specific response or emotion), that function can transfer to other stimuli related to B (e.g., to Stimulus C if C is equivalent to B, or even transform appropriately if C is in an opposite relation to B). These emergent behaviors happen without additional direct training, illustrating the generative power of relational learning. They are considered core aspects of human language and reasoning, enabling abstraction, analogy, and symbolic thought (Stewart and McElwee, 2009; Hayes et al., 2021).

From a developmental and functional perspective, RFT suggests that what we call “intelligence” is largely the repertoire of relational frames an individual has learned (Cassidy et al., 2016; Hayes et al., 2021). A person who can relate events in terms of temporal order can understand cause-effect; one who can relate perspectives (“I” vs. “you,” “here” vs. “there”) develops theory of mind and empathy; one who can relate items in terms of comparison can solve quantitative problems, and so on. These relational abilities build on more basic learned behaviors (like discrimination and conditioning), but introduce a qualitatively higher level of flexibility. Importantly, AARR is assumed to be trainable—through sufficient interaction and reinforcement, even complex relational patterns can be established. Indeed, educational programs based on training relational skills have been shown to increase measures of intelligence and cognitive aptitude (Cassidy et al., 2016). This view implies that an artificial system, too, could attain human-like cognitive versatility if it can be trained to exhibit AARR across a wide range of relations (Johansson, 2019a, 2020). In other words, a roadmap to human-level symbolic reasoning in AI would prioritize mechanisms for learning and applying relational frames, atop a foundation of operant learning capability.

In summary, RFT provides a behavioral account of cognition and symbolic reasoning, framing it as learned relational behavior. AARR encompasses skills like analogy-making, language understanding, and abstract problem-solving, which are precisely the capacities we seek in advanced AI (Hayes et al., 2021; Johansson, 2019a). The challenge for AGI research is how to implement these relational learning abilities in a machine. Machine Psychology addresses this by identifying necessary cognitive mechanisms (like representing relations and adjusting them based on feedback) and integrating them into an AI system. In the following sections, we turn to the Non-Axiomatic Reasoning System, which serves as the AI platform in which operant and relational learning principles are progressively realized.

## 4 The non-axiomatic reasoning system (NARS) architecture

The Non-Axiomatic Reasoning System (NARS) introduced by Pei Wang is designed to perform general-purpose reasoning under the realistic conditions of uncertainty, incompleteness, and limited computational resources (Wang, 2006, 2013). Unlike traditional AI systems that rely on fixed axioms or exhaustive knowledge, NARS is built on the assumption that an intelligent system must be able to adapt to its environment with incomplete information, while making the best use of finite time restrictions and limited memory capacity. This working assumption is referred to as the Assumption of Insufficient Knowledge and Resources (AIKR) (Wang, 1995, 2013). In practical terms, NARS does not presume any problem-solving domain to be closed or fully known; instead, it continuously updates its beliefs and priorities as new tasks and information arrive.

At the core of NARS is a cognitive logic with experience-grounded semantics, named Non-Axiomatic Logic (NAL) (Wang, 2013). NAL is a term logic that extends propositional and syllogistic reasoning to operate with uncertain judgments. Every statement in NARS is expressed as a term or a relationship between terms (e.g., a term can represent a concept like “apple” or a compound like “apple → edible”), and each statement is associated with a truth value reflecting the system's degree of belief in that statement based on past evidence. The truth value in NARS has two components: frequency $f:=\frac{w^{+}}{w}\in \left[0,1\right]$, representing the ratio of how often the statement has been observed to hold true in all observed, applicable cases, and confidence $c:=\frac{w}{w+1}\in \left[0,1\right)$, representing the reliability of that frequency value in light of future evidence, based on the total amount of evidence *w* = *w*^+^+*w*^−^ that supported or contradicted the statement (Wang, 2013; Hammer, 2022). This allows NARS to reason empirically in a quantitative sense: any inference it makes carries forward uncertainty from premises to conclusions, unlike logics based on true/false or discrete sets of truth values. For example, if NARS observed that in most cases *X* occurred and was followed by *Y*, and seldomly that *X* occurred without *Y* following, it will assign a high frequency to the implication “X ⇒ Y” even when the total amount of cases (and hence confidence as well), had been small. In contrast, if the total amount of both the positive and the negative cases together is high, confidence will also be high irregards of the frequency.

NARS supports a variety of inference rules covering deduction, induction, abduction, and analogy, all unified under the same truth-value scheme (Wang, 2013). Part of the logic, for instance, are syllogisms, which support all inference types: deducing conclusions from two premises, inductively generalizing from examples, abductively creating explanations, and finding analogies between relationships, using a common mechanism for evaluating how strongly conclusions follow. Because NARS was conceived as a model of “general” intelligence, its reasoning is not limited to deductive reasoning and supports hypothetical conclusions. It updates its beliefs in an incremental fashion: new evidence can strengthen or weaken a belief's truth value, and NARS can revise previous conclusions if they become untenable under new information (Wang, 2006, 2022). In effect, the system's knowledge is not static, but a dynamically evolving network of interconnected concepts and relations, aiming to capture on an abstract level how humans update their beliefs with experience.

Another defining feature of NARS is its approach to resource-constrained operation. The system maintains a concept memory where each concept acts as a container that aggregates the tasks (questions to answer or goals to achieve) and beliefs related to a particular term. NARS cannot “attend" to all concepts at all times and process them exhaustively; instead, it employs a priority-based scheduling mechanism that decides which tasks and belief to select next for inference, based on their urgency and importance (Wang, 1995; Hammer et al., 2023b). Each task and concept carries a priority value (or “budget”), and these are dynamically adjusted as tasks succeed, fail, or become more relevant. This ensures that with limited processing capacity, NARS focuses on what appears most promising or relevant at the moment, essentially realizing a form of attentional control. Because of this, NARS's reasoning process is non-deterministic and context-driven: the exact chain of inference can differ depending on which tasks are competing for attention, replicating how humans might solve the same problem in different ways, especially under time pressure (Wang, 2022). The benefit is robustness and adaptability: NARS is continually ready to handle new problems and can interleave different goals, rather than executing a fixed algorithmic plan for one problem at a time.

In summary, NARS provides a unified framework for reasoning and learning. It can accept new knowledge at any time (as input statements or sensory observations), incorporate it into its belief network, and use it to make inferences or guide actions. In fact, learning in NARS is not accomplished by a separate algorithm but is an intrinsic outcome of its inference process: whenever NARS derives a new implication or updates a truth value from evidence, it is effectively learning from its experience (Wang, 2022). This continuous learning blurs the line between reasoning (drawing conclusions) and learning (updating knowledge), and aligns it well with the needs of an autonomous AGI agent that must operate in real time in a changing world (Wang, 2019). In this respect, NARS provides a computational platform whose processes are consistent with how RFT understands human reasoning and learning: adaptive behavior shaped by consequences within a resource-limited system. From the perspective of psychology, one can see NARS's concepts and their relations as analogous to interconnected relational networks and their response strengths. The system's gradual adjustment of truth values is reminiscent of learning curves, and its priority mechanism echoes cognitive processes of attention and motivation.

For Machine Psychology, NARS offers a solid foundation because its design principles already resonate with psychological ones: effective action repertoires are acquired through experience, uncertainty is a fundamental aspect of cognition, and cognitive resources (attention, memory) are limited and must be managed. What NARS did not originally include, however, were specific facilities for operant conditioning or complex relational learning as studied in psychology. The next section introduces OpenNARS for Applications (ONA), a variant of NARS that extends it into the sensorimotor domain, making it possible to implement and test operant learning paradigms with NARS as the “brain” of an artificial agent.

## 5 OpenNARS for applications: sensorimotor reasoning in real time

**Figure 1:**
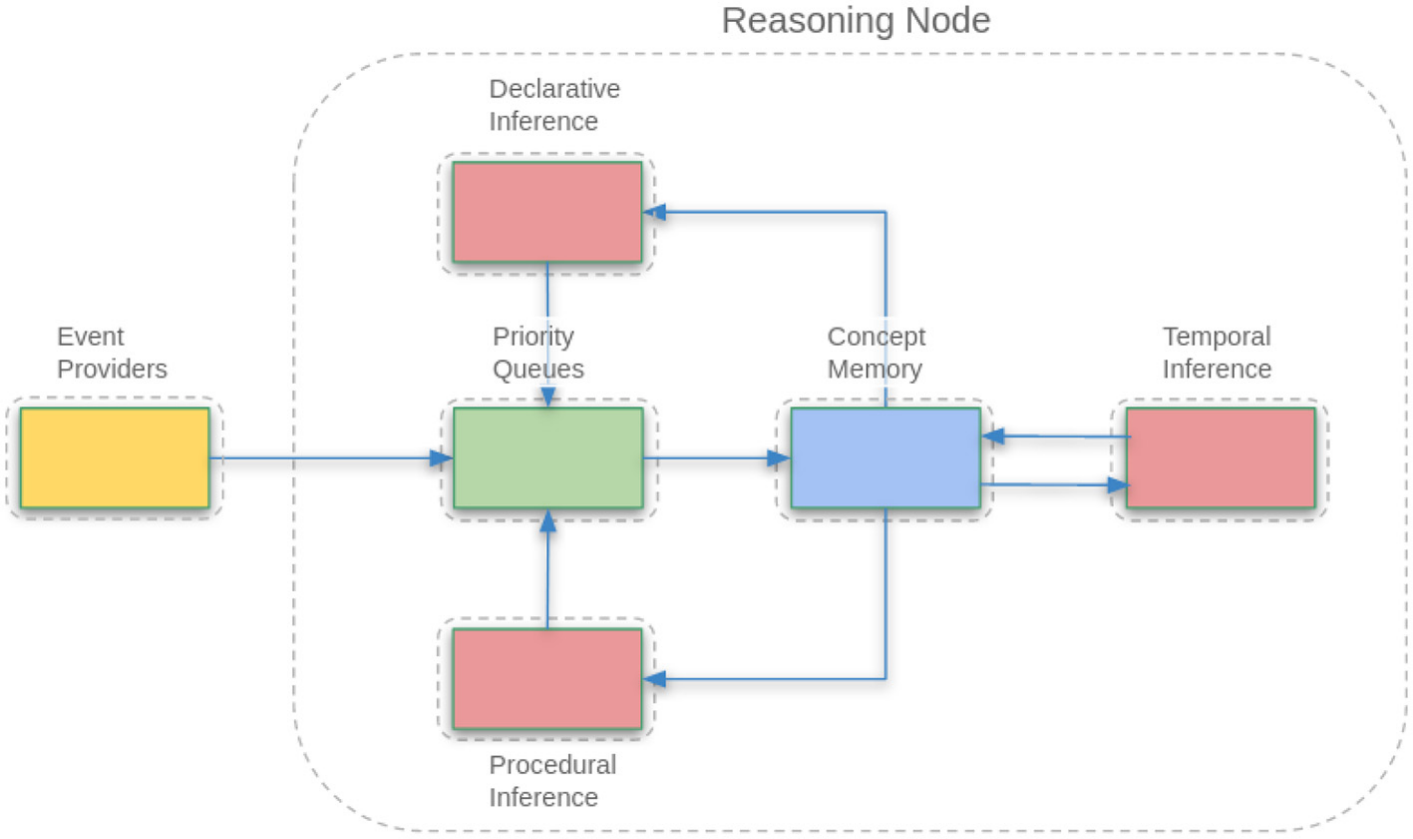
Architecture of OpenNARS for applications (ONA). Event providers supply sensory input to priority queues, which feed into concept memory. Three inference subsystems operate on concept memory: declarative inference for semantic knowledge, procedural inference for action selection and planning, and temporal inference for learning temporal dependencies. This architecture enables real-time sensorimotor reasoning while maintaining NARS's core reasoning capabilities.

OpenNARS for Applications (ONA) is an implementation of NARS tailored for embedded systems and mobile robotics (Hammer and Lofthouse, 2020) and other applications that demand real-time reasoning and updating. While the core reasoning engine of ONA remains Non-Axiomatic Logic, ONA introduces architectural refinements that enable more practical, real-time interactions with environments. One of its key features is the explicit separation into different inference pathways for declarative, temporal and procedural reasoning: this allows ONA to more specially treat sensory inputs and motor outputs represented as terms in the NARS language (Narsese), allowing the reasoning system to directly perceive and act in an environment (Hammer and Lofthouse, 2020; Johansson, 2024b). In essence, ONA endows NARS with “ears, eyes, and hands,” making it an embodied cognitive agent rather than just an abstract reasoner. The architecture of ONA is illustrated in Figure 1.

Several enhancements distinguish ONA from earlier NARS implementations:

- Event-driven control: ONA employs an event-driven control loop. While traditional NARS uses a stochastic “bag” of tasks that are processed often repeatedly and in turns, ONA instead prioritizes incoming events (new observations or tasks) and selects them once strictly based on the priority ranking. This suits dynamic environments where timing is critical (Hammer and Lofthouse, 2020). By focusing computational effort on new and relevant events, ONA improves responsiveness, much like an animal attending to a sudden stimulus.
- Sensorimotor vs. conceptual separation: ONA's design clearly separates sensorimotor inference from higher-level semantic inference. It has distinct subsystems for procedural reasoning about sensorimotor contingencies (e.g., what motor command to issue given a certain sensory situation) vs. reasoning on abstract knowledge (e.g., logical relations between concepts that are not tied to the present moment). This division allows the agent to manage real-time control and long-term reasoning in parallel without one overwhelming the other (Hammer and Lofthouse, 2020). For example, a robot controlled by ONA can maintain balance and avoid obstacles (sensorimotor tasks) while simultaneously deriving declarative knowledge, these represent different simultaneous inference activities that communicate through shared memory of concepts and are coordinates through the priority queue structures.
- Resource management and memory: ONA intensifies NARS's focus on resource management. It implements efficient data structures for events and concepts, and uses a combination of FIFO queues and priority queues to ensure important information is processed first. Mechanisms like forgetting (discarding and lowering priority of seldomly, not recently, used concepts) are used to keep the system scalable over long run-times, analogous to how animals gradually forget irrelevancies. The system's memory is thus self-organizing, aiming to devote just enough attention to each piece of knowledge based on context.
- Continuous learning in environment: ONA can interface with sensors and effectors, and is able to learn procedural knowledge (via inductive inference) that can guide its actions. In NARS, an action is represented as an operator (e.g., ^pressButton could be an operator). ONA can learn conditions under which executing an operator will achieve a desired result, represented as temporal implications in its knowledge base (Johansson, 2024b). For instance, the system might learn a temporal implication like “(see light and have lever) ⇒ [press lever] achieves goal,” with a truth value that increases as it experiences success. This is essentially how ONA implements operant conditioning at the cognitive level: it uses temporal and procedural inference to model the outcomes of its actions in different situations. The system updates these beliefs as it gains more experience, thereby improving its future decision-making.

ONA has been demonstrated to be capable of controlling autonomous robots and simulated agents (Hammer et al., 2023b). In one example, Hammer et al. (2023b) used ONA to control a ROS-based robot, showing that it can integrate multiple sensor inputs (e.g., camera, touch sensors) and decide on motor actions in real time. The event-driven and resource-bounded nature of ONA allowed the robot to operate continuously and adapt to unexpected mission-critical changes (e.g. a bottle tipping over or getting moved by a human operator) without reprogramming. These features are crucial for applying Machine Psychology experiments, which often require the agent to be “left on its own” to discover solutions via feedback.

In our context, ONA provides the testbed for all the empirical studies of Machine Psychology. It embodies NARS's reasoning within an agent that perceives and acts. When an operant conditioning experiment is designed, stimuli and reinforcement contingencies are specified in a simulated environment, and ONA uses its sensorimotor reasoning to learn the task without any prior knowledge of the correct behavior (Johansson, 2024b). Similarly, for training on relational tasks, its capability to represent abstract symbols and contextual cues as sensorimotor events is exploited (e.g., a “context cue” like SAME vs. OPPOSITE can be given to the system as a special perceptual input that it treats as part of the state, see Section 9). By treating these experimental tasks as just another set of inputs and outputs, we ensure that it is not hard-coded for any particular task. In order to succeed, it must genuinely learn and reason, just like an animal would in a psychology experiment.

In summary, ONA realizes NARS as an embodied agent that can engage in realistic learning tasks. It maintains the core strengths of NARS (integrated uncertain reasoning, adaptive memory) while adding the practical capabilities needed to interact with an environment (Hammer and Lofthouse, 2020; Hammer, 2022). This makes it feasible to translate psychological paradigms, such as a Skinner box or a matching-to-sample task, directly into a form NARS can participate in. The following Sections 6–9 detail how a series of such tasks were implemented and what ONA/NARS was able to achieve, hereby we will start from various forms of operant behavior and identity matching, toward demonstrations of Arbitrarily Applicable Relational Responding.

## 6 Operant conditioning with NARS (Study I)

The first empirical challenge for Machine Psychology was to show that NARS can learn through operant conditioning, i.e., by adjusting its behavior based on reinforcing consequences over time. This was addressed by Johansson (2024b), who evaluated NARS/ONA on a set of fundamental operant learning tasks. In brief, the experimental setup consisted of three tasks of increasing complexity (simple discrimination, reversal learning, and conditional discrimination), each using 2–4 visual stimuli (colored lights or shapes) presented in a simulated Skinner box environment. Trials were organized in blocks of 6, with a continuous reinforcement schedule (each correct response producing a reinforcing consequence). Performance was measured as percent correct per block. States were encoded as Narsese statements representing the currently visible stimuli, and actions as NARS operators (e.g., ^select-left, ^select-right).

In these experiments, ONA was placed in the simulated environment: the system could choose among certain actions (e.g. press a left or right button) in the presence of particular stimuli, and it would receive feedback indicating success (reinforcing consequence) or failure for each choice. No prior knowledge of the correct behavior was given; the system had to discover which actions were appropriate through trial and error. NARS's inductive inference and temporal reasoning capabilities enabled it to discover action–outcome contingencies autonomously: by observing temporal co-occurrences between actions and outcomes, NARS could form hypotheses about which actions lead to reinforcement in which contexts, without any such contingencies being pre-programmed.

**Figure 2:**
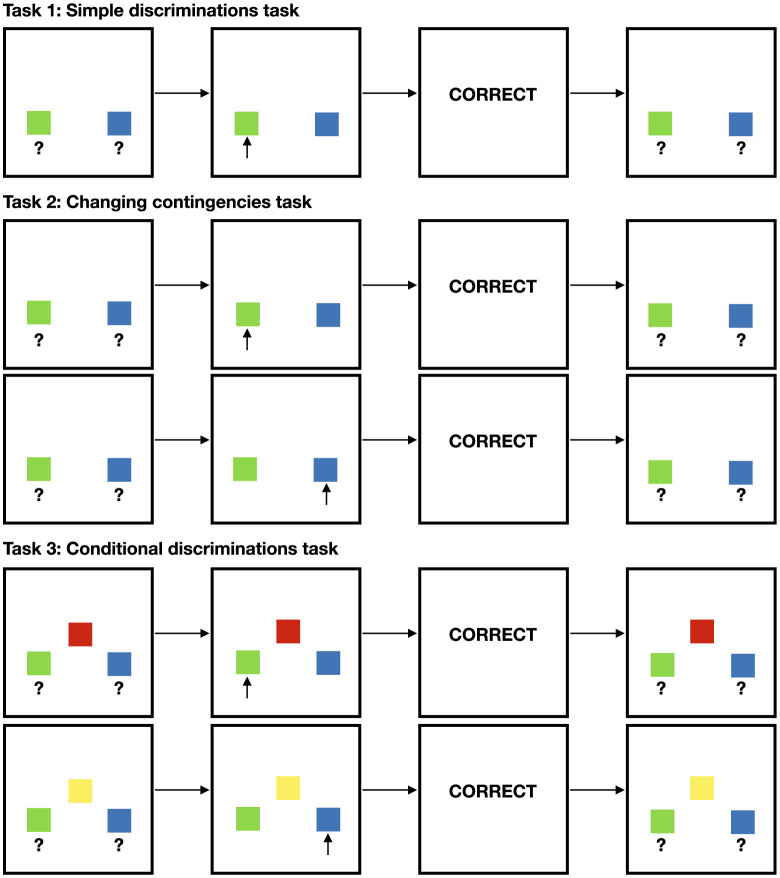
The three operant conditioning tasks used in Study I. Task 1 (simple discrimination): the agent must learn to select the correct stimulus (green) to receive reinforcement. Task 2 (changing contingencies): after learning an initial discrimination, contingencies are reversed, requiring the agent to adapt. Task 3 (conditional discrimination): a contextual cue (red or yellow) determines which comparison stimulus is correct, requiring conditional reasoning.

**Figure 3:**
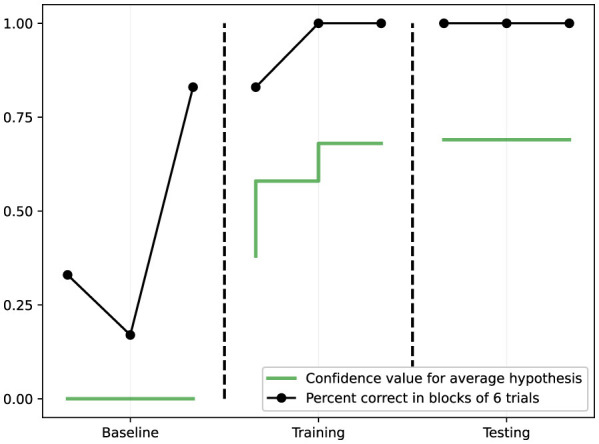
Results from Task 1 (simple discrimination). The black line shows percent correct responses in blocks of 6 trials across baseline, training, and testing phases. The green line shows the confidence value for the learned hypothesis. Performance rapidly improves during training and remains at 100% during testing, while hypothesis confidence increases steadily.

**Figure 4:**
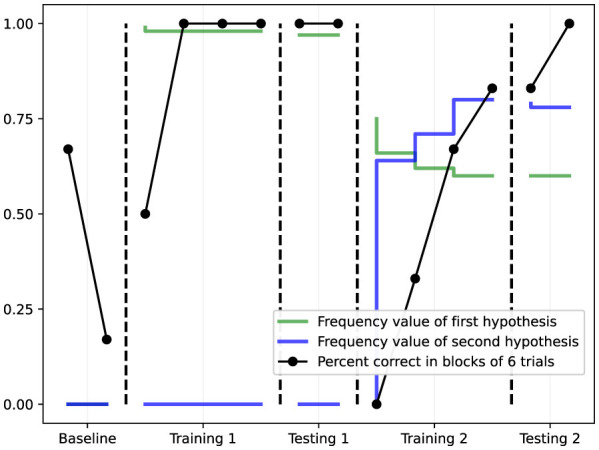
Results from Task 2 (changing contingencies/reversal learning). Performance (black line) reaches 100% during Training 1, drops when contingencies are reversed (Training 2), then recovers. The green and blue lines track the frequency values of the first and second hypotheses, showing how NARS updates its beliefs when the environment changes.

**Figure 5:**
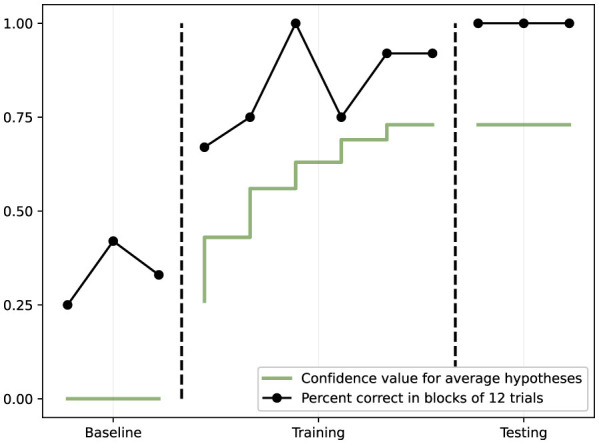
Results from Task 3 (conditional discrimination). Performance (black line) improves during training and reaches 100% during testing. The green line shows confidence in the conditional matching hypothesis, demonstrating that NARS successfully learned the context-dependent rule.

Three operant tasks of increasing complexity were used (Johansson, 2024b), as illustrated in Figure 2:

- Simple discrimination: the agent sees a single stimulus (e.g. a colored light) and must learn which one action leads to reinforcement. For example, when a green light is present, pressing the left button might yield a reinforcing consequence while pressing right does nothing, and vice versa for a blue light. NARS (via ONA) starts by exploring actions randomly. It soon observes that, say, in context “green light,” action “^select-left” is followed by reinforcement, and forms a procedural belief linking the two. In the experiment, NARS rapidly learned the correct response for each light. After the blocks presented within six trials, it achieved 100% correct responses, and it retained this performance in subsequent test trials with the same stimuli but no feedback (extinction test). Internally, NARS's confidence in the action–outcome implications (e.g. “green and left ⇒ goal achieved”) rose significantly from initial guesses (around confidence 0.3) to a high level (around 0.7) as it accumulated evidence. This corresponds to the system “figuring out” the contingency much like an animal would. Johansson (2024b) reports that NARS's learning curve in this task qualitatively resembles that of a lab animal—a sharp transition from chance level to near-perfect behavior once the contingency is discovered (see Figure 3).
- Changing contingencies (reversal learning): in this task, the correct action for a given stimulus was reversed halfway through the experiment. For instance, initially green → left is correct and blue → right is correct; after a number of trials, the rule flips (now green → right, blue → left yield reinforcement). This tests the system's adaptability: can it unlearn a previously successful behavior and learn a new one when the environment changes? NARS successfully navigated this challenge (Johansson, 2024b). It showed a brief performance drop when contingencies were switched—as expected, it continued with the old response a few times with no reinforcement—but within a short number of trials it adjusted and reached high success rates again. The system accomplished this by updating its implications: when past reliable actions stopped yielding reinforcing consequences, their truth values (frequency/confidence) decreased, and through further exploration NARS identified the new correct actions, forming new implications (or strengthening previously weak ones that corresponded to the alternative action). Essentially, NARS “noticed” the environment contingency had shifted and re-learned accordingly, demonstrating cognitive flexibility akin to an animal in a reversal learning experiment (see Figure 4).
- Conditional discrimination: this more complex task introduces two stimuli simultaneously and a context-dependent rule. For example, a matching-to-sample setup: the agent is shown a sample stimulus (e.g., a color) and two option stimuli (left and right), and it must choose the option that matches the sample. One trial might present green as a sample with a green on left and blue on right—the correct choice is left (match green with green); another trial might show blue as sample with green vs. blue options—correct is right (match blue with blue). This is conditional because the correct action (left or right) depends on the sample's identity. NARS was able to solve such tasks as well, using its ability to represent conjunctions of conditions and outcomes. ONA encodes each trial's state (sample = X, left = Y, right = Z, etc.) as a series of Narsese statements, and a reinforcement signal as a goal achievement event. During training, NARS gradually forms a conditional implication in memory like: “if (sample and left option are the same) and I choose left, then goal”—in Narsese this is a compound implication linking the condition of a match to the action and the reinforcing outcome. Johansson (2024b) notes that NARS achieved high accuracy in the conditional discrimination task after sufficient training, and importantly, it could handle combinations not explicitly seen before, indicating it truly learned the general principle. By the end of training, its derived “matching” hypothesis had a strong confidence, and test trials confirmed that it applied this hypothesis correctly to novel pairs (see Figure 5).

Overall, the results from these operant conditioning experiments validate that NARS (with ONA) can indeed replicate basic animal learning processes (Johansson, 2024b). It can form and revise stimulus–response relations based on reinforcement, pursue reinforced actions, and avoid non-reinforced ones, all without any hardcoded task-specific algorithms. The underlying NARS mechanisms that enable this include temporal inference (to correlate an action with a subsequent reinforcing consequence in time) and procedural learning (representing an action selection as an implication to achieve a goal). In psychological terms, NARS's behavior illustrates selection by consequences—effectively, the system “selects” (increases priority of) those internal action plans that lead to reinforcement, a parallel to how evolution selects successful behaviors. The experiments also showcased NARS's resilience to change (reversal learning) and its capacity for conditional reasoning when multiple cues are involved.

From an AGI standpoint, these findings are significant. They demonstrate that even a logic-based AI system like NARS, which is very different from typical reinforcement learning algorithms (Hammer, 2022), can exhibit operant learning when equipped with the right representational framework (Johansson, 2024b). This builds confidence that we can proceed to layer more complex cognitive skills atop this operant foundation. Just as infants first learn sensorimotor contingencies before they learn language, NARS first needed to master basic learning from reinforcing consequences. With that in place, the next step in Machine Psychology was to explore generalization—can NARS learn an abstract concept from examples and apply it beyond the training instances? The following section examines the second study, which tackled this question through generalized identity matching.

## 7 Generalized identity matching with NARS (Study II)

Generalization—the ability to apply learned knowledge to new, unseen situations—is a critical hallmark of intelligence. In humans and animals, one form of generalization studied in learning psychology is generalized identity matching. After learning to match specific stimuli by identity (e.g., given a sample stimulus, choose the identical one among options), a subject is tested with entirely new stimuli to see if it can still perform the matching task correctly. Success indicates that the subject has abstracted the concept of “sameness” or identity, not just memorized particular pairings. Young children and some animals can achieve this after sufficient exemplars, though it may be challenging for non-humans without extensive training or contextual cues. The question for Machine Psychology was: can NARS acquire an abstract identity relation and demonstrate one-shot generalization to novel stimuli? Johansson et al. (2023) addressed this by implementing a generalized matching-to-sample task in NARS/ONA.

In brief, the experiment used a matching-to-sample procedure with two training stimuli (e.g., green and blue) and two novel test stimuli (e.g., red and yellow). Trials were organized in blocks of 12. During each trial, ONA was presented with a sample stimulus and two comparison stimuli, and had to select the comparison matching the sample to receive a reinforcing consequence (continuous reinforcement schedule). States were encoded as Narsese statements specifying the sample and comparison features, and performance was measured as percent correct per block. The critical dependent variable was accuracy on novel stimuli never encountered during training.

**Figure 6:**
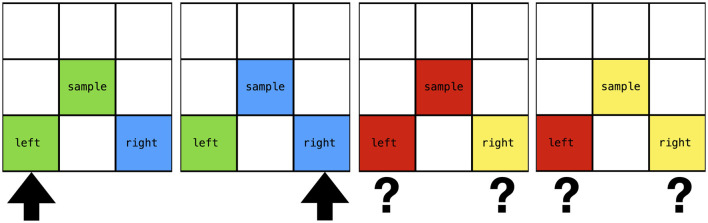
The generalized identity matching-to-sample task (Study II). Left panels show training trials with green and blue stimuli where the agent learns to select the comparison that matches the sample. Right panels show generalization test trials with novel stimuli (red and yellow) that were never reinforced during training. Question marks indicate that the correct response must be derived from the learned general rule.

The experimental setup in NARS mirrored a typical identity matching task used in animal research (see Figure 6). During training, ONA was presented with a sample (a particular symbol or feature) and two comparison options, one of which was identical to the sample. NARS had to choose the matching one to receive reinforcement (similar to the conditional discrimination described earlier, but across multiple exemplars). For example, one training trial might show “Sample: Shape A; Left: Shape A; Right: Shape B”—the correct choice is left. Another trial could be “Sample: Shape B; Left: B; Right: A”—correct is left in that case (assuming left holds the matching stimulus). A variety of stimuli (shapes, colors, etc.) were used in training so that no single physical stimulus was the basis of learning; rather, the relation “same-as” was the common factor. NARS was equipped with the variable introduction capability, meaning it could form rules with placeholders (variables) that stand for “any object” in certain positions. This is analogous to how a human might form the rule “select the option that matches the sample” without tying it to a specific sample identity.

During training, NARS went through multiple phases: baseline (pre-learning) tests to confirm no innate bias, a training phase with feedback on several specific sample-comparison sets, and then a critical generalization test with novel stimuli. Johansson et al. (2023) report that NARS's performance improved rapidly during training, reaching high accuracy on the trained pairs, and—importantly—it performed perfectly on the novel pairs in the generalization test. This indicates that NARS had indeed abstracted the identity-matching rule. Through its inference process, NARS autonomously derived a general implication with a variable. This was not pre-programmed but emerged from NARS's inductive reasoning over its accumulated experience. The derived rule, expressed in Narsese, took the form:


  (< (sample * #1) --> (loc * slot)> &/
    < (left * #1) --> (loc * slot)> &/
    < ({SELF} * (sample * left)) --> ^match>) =/> G
 


**Figure 7:**
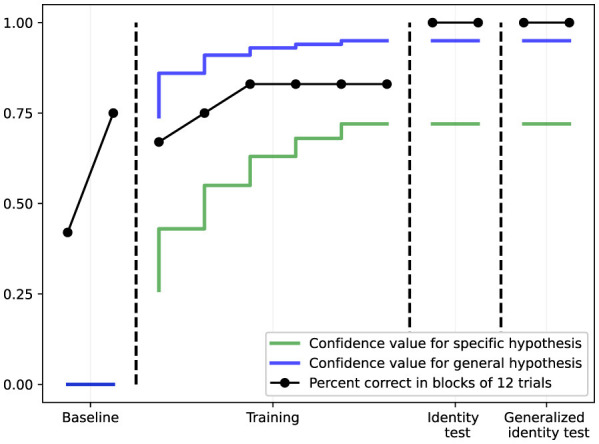
Results from the generalized identity matching study (Study II). The black line shows percent correct in blocks of 12 trials across baseline, training, identity test (with trained stimuli), and generalized identity test (with novel stimuli). The green and blue lines show confidence values for specific and general hypotheses respectively. NARS achieves 100% success rate on both the identity test and the critical generalized identity test, demonstrating successful abstraction of the matching rule.

In plain language, this reads: “If the sample and the left comparison share the same feature value (whatever it may be), then selecting the left comparison as a match achieves the goal.” The #1 is a variable that can bind to any stimulus identity, making the rule general rather than tied to specific stimuli (Johansson et al., 2023). The truth value (particularly the confidence) of this abstract hypothesis increased with training experience, from near zero to a substantially high value, reflecting NARS's growing belief in the general rule. By the end of training, NARS effectively trusted the implication that “choosing the same leads to reinforcement,” and when presented with never-seen-before stimuli in the test (e.g., a new shape or symbol as sample and two options), it relied on that generalized implication to make its choice. As a result, it chose correctly even without any direct reinforcement for those new stimuli—a clear demonstration of generalization (see Figure 7) (Johansson et al., 2023).

This experiment highlights the role of the variable introduction mechanism in NARS. Variable terms allow NARS to find common structure across experiences and induce a rule that is not tied to specific constants (Wang, 2013; Johansson et al., 2023). In psychological terms, NARS acquired a concept of identity matching, analogous to how a child learns a general principle like “an object is the same as itself” or “match same with same.” The success of NARS in this task is notable because it did not involve any specialized neural architecture or backpropagation through examples—it emerged from NARS's logical induction capabilities supplemented by the ability to introduce a variable to generalize a pattern. This shows that even in a symbolic reasoning system, with appropriate design, one can achieve behavior functionally similar to what is observed in animal learning experiments concerning concept formation (Johansson et al., 2023).

Johansson (2024a) further explored NARS's generalization capacity by testing a variant of the task involving comparative relations (Hammer et al., 2023a). They enabled an alternative mode where NARS could use a comparative reasoning operator to, for example, pick an item that is brighter or larger (a non-arbitrary relation) rather than identical. This was to see if NARS could also generalize a “relative” rule. They found that NARS could indeed learn rules like “pick the larger one” in a similar fashion when provided with comparative feedback (Johansson, 2024a). Although this goes beyond pure identity matching, it reinforces the point that NARS's learning apparatus can capture different kinds of relations by induction, given the right feedback and the right internal representation (variables, in this case).

In summary, generalized identity matching in NARS was achieved and serves as a proof-of-concept that the system can learn an abstract relational rule from examples. This result is an important milestone on the Machine Psychology roadmap: it corresponds to moving from simple operant associations to a form of symbolic generalization, arguably a prerequisite for higher cognition (Johansson et al., 2023). The ability to generalize is what allows an AI to avoid the brittleness of pure memorization. For AGI, it means the system can tackle novel situations using previously acquired knowledge—exactly what we expect of human intelligence. With identity matching under its belt, the next step was to push NARS's relational learning further into the realm of derived relations and equivalence, which we address in the following section on functional equivalence.

## 8 Functional equivalence with NARS (Study III)

As cognitive abilities grow, an intelligent agent not only recognizes when two stimuli are physically identical but can also learn when disparate stimuli are effectively equivalent because they serve the same function or lead to the same outcome. In psychology, functional equivalence refers to the grouping of stimuli into classes such that the individual treats all members of a class interchangeably in certain contexts, due to their shared consequences or meaning (Sidman, 2000). For example, a person might consider two different doorbells equivalent if pressing either yields the same sound and opens the same door—functionally, they are substitutes. Functional equivalence, as modeled here, is related to but not identical to full Sidman stimulus equivalence; it captures transfer of function across stimuli with shared consequences but does not implement reflexivity, symmetry, and transitivity in the formal Sidman sense. Nevertheless, it plays a role in concept formation (different stimuli representing the same concept) and serves as a stepping stone toward more complete equivalence relations.

In the context of NARS, functional equivalence was studied by Johansson et al. (2024) to see if the system can spontaneously transfer learning from one stimulus to another when they share a common outcome. The experiment (detailed in Johansson, 2024a) involved training NARS on a set of stimulus–response relations and then testing whether it generalizes new such functional relations to stimuli that were never directly paired before, purely because those stimuli had equivalent roles in the past interactions. In brief, the study used four visual stimuli (A1, A2, B1, B2) and four response options (R1–R4). Trials were organized in blocks of 12 across three phases (training, retraining, and testing), with a continuous reinforcement schedule during training phases and no feedback during the critical test phase. Performance was measured as percent correct per block, and internal confidence in equivalence hypotheses was tracked throughout.

**Figure 8:**
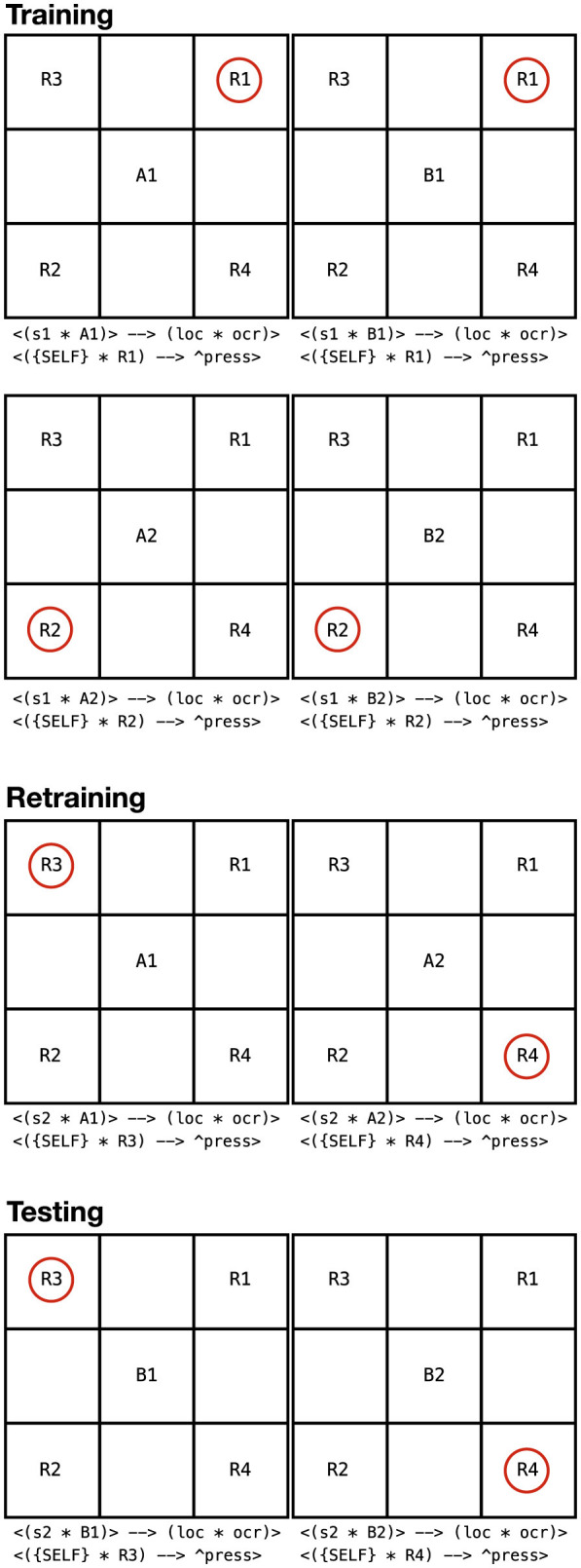
The functional equivalence task (Study III). Training phase: stimuli A1 and B1 both require response R1, while A2 and B2 require R2, establishing functional equivalence classes. Retraining phase: Only A1 and A2 are retrained to new responses (R3 and R4 respectively). Testing phase: B1 and B2 are tested with the new responses. If NARS has formed equivalence classes, it should derive that B1 now requires R3 (like A1) and B2 requires R4 (like A2). Circled responses indicate correct selections.

The procedure consisted of three phases (see Figure 8):

- Phase 1 (training): NARS was trained on several fixed stimulus-response mappings. Using a simplified example: suppose stimuli A1 and B1 both required pressing button R1 to receive reinforcement, while A2 and B2 both required pressing button R2 for reinforcement. During this phase, ONA experiences contexts like “Sample A1 → press R1 → reinforcement” and separately “Sample B1 → press R1 → reinforcement,” likewise for A2 and B2 with R2. After training, NARS has learned these specific four functional relations (A1 → R1, B1 → R1, A2 → R2, B2 → R2). The performance reached near 100% on these trained pairs, indicating solid learning (just as in the operant tasks). Importantly, from NARS's perspective, A1 and B1 both predict the same reinforced action R1, and similarly A2 and B2 predict action R2. This lays the ground for equivalence: A1 and B1 share a functional property (“pressing R1 produces reinforcement”), likewise A2 and B2 share another.
- Phase 2 (re-training with new outcomes): now the contingencies were changed for one member of each pair. For example, A1 and A2's required responses were switched: A1 now requires a different button (say R3) to receive reinforcement, and A2 requires R4—entirely new responses that B1 and B2 have never encountered. B1 and B2's mappings remain as before during this retraining (B1 → R1, B2 → R2). NARS was trained on these new A1 and A2 mappings. As expected, it adapted quickly: within a few trials it reached 100% correct for A1 → R3 and A2 → R4 (demonstrating flexibility similar to the reversal learning earlier). At this point, A1's function changed (it now correlates with R3 not R1), and A2's function changed (now R4 not R2). B1 and B2 were still associated with R1 and R2 respectively, since they were not retrained in this phase.
- Phase 3 (testing for derived equivalence): finally, NARS was tested on B1 and B2 with the new responses that A1 and A2 had been retrained to. In other words, without any direct training, the system was presented with B1 and given the opportunity to press R3, and with B2 given the chance to press R4—these combinations were never reinforced or shown previously. If NARS presses R3 for B1 (and R4 for B2) reliably, it indicates that NARS has inferred B1 must now go with R3 because B1 was functionally equivalent to A1, and A1 was switched to R3; similarly B2 to R4 via A2. Johansson et al. (2024) report exactly this result: NARS performed at 100% correct on those derived stimulus-response relations (B1 → R3, B2 → R4) in the test phase, without any direct feedback guiding them. This demonstrates that NARS had formed an equivalence class (A1 with B1, and A2 with B2). When A1's “meaning” (required action) changed, NARS propagated that change to B1 automatically, treating B1 as interchangeable with A1 in terms of the outcome.

**Figure 9:**
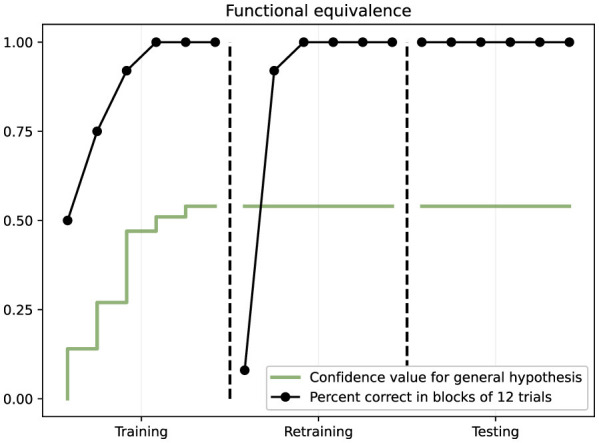
Results from the functional equivalence study (Study III). The black line shows percent correct in blocks of 12 trials across training, retraining, and testing phases. The green line shows confidence in the general equivalence hypothesis. Performance initially drops during retraining when contingencies change for A stimuli, then recovers. Critically, NARS achieves 100% correct on the testing phase for B stimuli without direct training, demonstrating successful transfer of function through derived equivalence.

From NARS's internal perspective, what happened is that during Phase 1 it acquired implications suggesting A1 and B1 both imply the goal via R1, and A2 and B2 via R2. Through its inference process, NARS established hypotheses of equivalence such as “A1 ⇔ B1” (and A2 ⇔ B2) meaning A1 and B1 are functionally related by yielding the same result. In Phase 2, when A1 was related to R3, NARS updated its relational network: now A1 implies goal via R3. Given A1 and B1 were linked, NARS could derive that B1 might also imply goal via R3 (mutual entailment of their equivalence relation). So by Phase 3, the highest-confidence action for B1 would have shifted to R3 due to that reasoning, and indeed NARS chose R3 for B1 with high confidence. The thesis notes that the average confidence in the hypotheses “A1 ⇔ B1” and “A2 ⇔ B2” increased steadily during training, indicating NARS was building up these equivalence relations quantitatively (Johansson, 2024a). Specifically, after Phase 1 the confidence was already above 0 (perhaps ~0.3), and by the end of Phase 2 it rose to about 0.54 on average for those equivalence hypotheses. This growing confidence shows NARS accumulating evidence that A1 and B1 go together (since both were correlated with R1 originally, then A1 changed but that change was noticed and B1's lack of change became evidence to support an inference). The results are shown in Figure 9.

In sum, functional equivalence with NARS was achieved. The system grouped stimuli by their outcomes and then generalized a change across members of a group, a hallmark of flexible adaptation (Johansson et al., 2024). Psychologically, this parallels how people can transfer learning: e.g., if two words mean the same thing in different languages, learning something new about one word (a new implication or usage) can transfer to the other. For AGI, this capability is crucial—it means the AI can form internal categories (equivalence classes) and treat new information about one element of a category as applying to others in that category. It's a step toward symbolic reasoning, where symbols in the same class are substitutable.

One can consider this result an instance of combinatorial entailment and transformation of function in RFT terms: A1 and B1 were mutually entailed as equivalent; when A1's relation to an outcome changed (function transformation), B1 accordingly changed its relation to that outcome (the function transformed across the equivalence relation). The NARS mechanisms introduced to facilitate this included a specialized representation for equivalence (Johansson et al., 2024) or simply the chain of implications that allowed NARS to derive new links. Specifically, they added what the thesis calls the “mechanism of functional equivalence” on top of the prior mechanisms (temporal/procedural reasoning and variable introduction). This involved allowing NARS to form higher-order implications (in two directions) that if two stimuli lead to the same consequence, it can postulate an equivalence relation. In implementation, this might be done by rules that detect patterns like “X → Z and Y → Z” and produce a hypothesis “X ⇔ Y” (where ⇔ might be realized via a pair of mutual implications in NAL). Given NARS's logical nature, such a hypothesis is then subject to confirmation by further evidence, which is consistent with the confidence gradually increasing as more support accumulates.

Having demonstrated functional equivalence, the Machine Psychology program moved to its most ambitious test: arbitrarily applicable relational responding (AARR), including arbitrary relations like “same” and “opposite” that require contextual cues. The next section reviews how NARS was extended to handle that level of relational complexity.

## 9 Arbitrarily applicable relational responding in NARS (Study IV)

The culmination of the Machine Psychology research program was to show that NARS can model AARR (as introduced in Section 3), going beyond simple equivalences to train and derive relations that are explicitly contextual—where whether two stimuli are treated as “the same” or “opposite” depends on a contextual cue.

Johansson (2019a) conceptually introduced the challenge of implementing AARR in an AGI system, and Johansson (2025) outlined a comprehensive framework for realizing AARR in NARS through the introduction of *acquired relations*—a mechanism added to NARS that provides explicit representations of learned relational structures, enabling the system to derive novel relations via mutual and combinatorial entailment. This framework was subsequently validated empirically by Johansson et al. (2025), who demonstrated same/opposite relational responding in NARS using a matching-to-sample paradigm. In brief, the study used a matching-to-sample procedure with contextual cues (SAME and OPPOSITE) across three phases: pretraining on relational properties using abstract stimulus sets (XY, YZ), relational network training with novel stimuli (AB, AC pairs), and derived relational testing with untrained stimulus combinations (BC pairs). Trials were organized in blocks of 16, with continuous reinforcement during training and no feedback during testing. Performance was measured as percent correct per block, and internal confidence in mutual and combinatorial entailment hypotheses was tracked throughout.

### 9.1 Acquired relations and relational naming

The key theoretical innovation enabling AARR in NARS was the introduction of *acquired relations* (Johansson, 2025). This is a mechanism introduced for the Machine Psychology research program—not an inherent capability of the base NARS architecture—that allows the system to abstract relational patterns from sensorimotor interactions into explicit relational hypotheses. Concretely, the mechanism works by detecting when a consistent relational pattern has been reinforced across matching-to-sample trials and generating an explicit relational statement that names the relation. When NARS learns through the matching-to-sample procedure that selecting stimulus B1 when presented with sample A1 under the “SAME” context leads to reinforcement, it not only forms a procedural contingency but also abstracts an explicit relational representation:


 
   < (A1 * B1) --> SAME>.
 


This explicitly named relational form enables NARS to respond to the relation itself, supporting novel relational derivations. NARS's inference rules were extended to handle combining these relations, allowing it to perform the relational entailments central to RFT: if NARS knows SAME(A1,B1) and SAME(A1,C1), it can infer SAME(B1,C1) through combinatorial entailment. Similarly, mutual entailment allows NARS to infer that if A1 is SAME as B1, then B1 is SAME as A1.

### 9.2 Same/opposite relational responding

The empirical validation of AARR in NARS focused on contextual control with two relational frames: Same and Opposite. In human RFT experiments, these relational frames can be established and then used to test whether subjects derive new relations based on the trained frames (Roche et al., 2000). The unique challenge is that “Opposite” frames require deriving inverted relations—if A is opposite to B, and B is opposite to C, then A is same as C.

**Figure 10:**
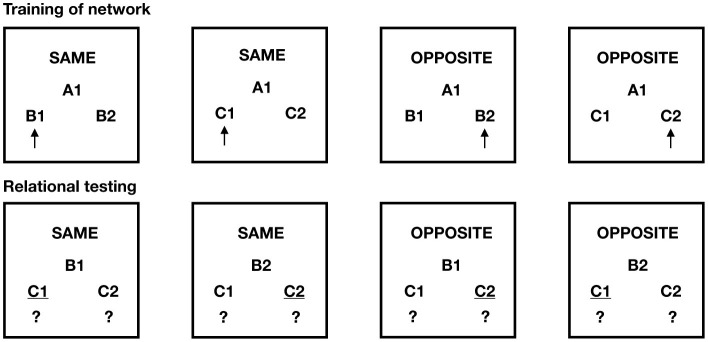
The AARR matching-to-sample task with SAME and OPPOSITE contextual cues (Study IV). Training of network: the agent learns that under the SAME cue, A1 goes with B1 and C1; under the OPPOSITE cue, A1 goes with B2 and C2. Relational testing: Novel combinations test whether NARS can derive untrained relations (e.g., given SAME with B1 as sample, does it select C1? Given OPPOSITE with B1, does it select C2?). Question marks indicate derived relations that must be inferred.

**Figure 11:**
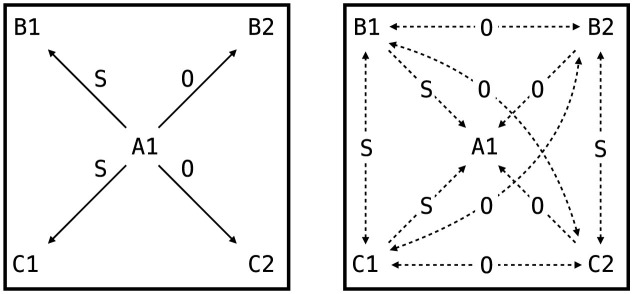
Relational network structure in the AARR study. **(Left panel)** trained relations (solid arrows) connecting A1 to B1, B2, C1, and C2 via Same (S) and Opposite (O) frames. **(Right panel)** The complete network including derived relations (dashed arrows) that emerge through mutual and combinatorial entailment. For example, if A1 is SAME as B1 and SAME as C1, then B1 and C1 are derived to be SAME; if A1 is OPPOSITE to B2, and B2 is SAME as C2, then A1 is derived to be OPPOSITE to C2.

Johansson et al. (2025) implemented and validated this scenario using a matching-to-sample procedure with contextual cues for “SAME” and “OPPOSITE” (see Figure 10). The experiment consisted of three phases:

- Phase 1 (pretraining): NARS was explicitly trained on the properties of mutual and combinatorial entailment for both SAME and OPPOSITE frames. This included training symmetrical relations (if X → Y under SAME, then Y → X under SAME) and transitive relations (if X → Y and Y → Z under SAME, then X → Z). For OPPOSITE, NARS learned that opposition is symmetric (if X opposite Y, then Y opposite X) and that combining two opposite relations yields sameness (if X opposite Y and Y opposite Z, then X same Z). NARS rapidly acquired these relational principles, achieving 100% accuracy by the end of pretraining (Johansson et al., 2025).
- Phase 2 (relational network training): using novel stimulus sets (AB and AC pairs), NARS was trained on a relational network where A1 was established as SAME with B1 and C1, while A1 was OPPOSITE to B2 and C2 (see Figure 11, left panel). Through matching-to-sample trials with reinforcement, NARS formed internal acquired relations representing these explicitly trained relationships. Performance reached 100% correct during this phase.
- Phase 3 (derived relational testing): the critical test phase presented stimulus pairs (BC) that were never directly trained. Without any reinforcement feedback, NARS was tested on whether it could derive the correct relations between B and C stimuli based on their trained relations to A stimuli. For example: given that B1 is SAME as A1, and C1 is SAME as A1, NARS should derive that B1 is SAME as C1 (combinatorial entailment). Similarly, given that B1 is SAME as A1, and C2 is OPPOSITE to A1, NARS should derive that B1 is OPPOSITE to C2. Critically, NARS also needed to derive that B1 is SAME as C1 when both were trained as OPPOSITE to A2—combining two opposite relations to yield sameness.

Figure 11 illustrates the trained relations (solid arrows) and the derived relations (dashed arrows) that emerge through mutual and combinatorial entailment. The results demonstrated that NARS achieved perfect accuracy (100%) in the derived relational testing phase, correctly inferring all untrained BC relations (Johansson et al., 2025). This performance significantly exceeded chance (50%), confirming that relational responding was driven by previously internalized relational structures rather than random guessing.

**Figure 12:**
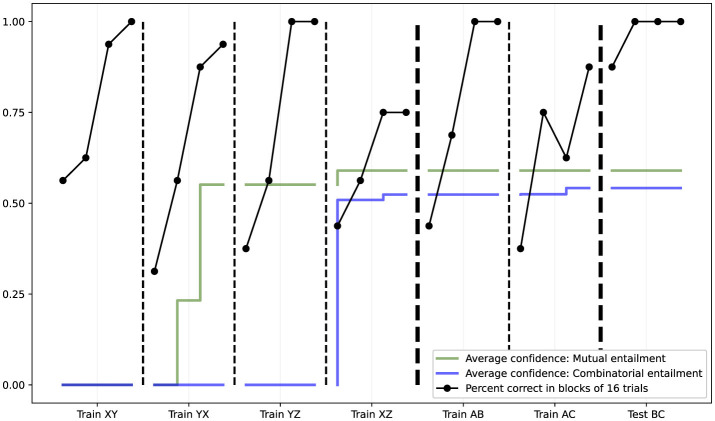
Results from the AARR study (Study IV). The black line shows percent correct in blocks of 16 trials across multiple training phases (Train XY, YX, YZ, XZ, AB, AC) and the critical Test BC phase. The green and blue lines show average confidence values for mutual entailment and combinatorial entailment hypotheses respectively. Performance improves through training, and critically, NARS achieves 100% accuracy in the Test BC phase, demonstrating successful derivation of untrained relations through combinatorial entailment.

Internal confidence metrics tracked throughout the experiment showed strong internalization of both mutual and combinatorial entailment hypotheses (see Figure 12). By the end of Phase 2, confidence values for both types of entailment had stabilized at high levels, providing the internal support necessary for successful derived responding in Phase 3.

### 9.3 Implications for symbolic cognition

This demonstration is significant because it shows NARS handling two intertwined relational contexts simultaneously—SAME and OPPOSITE—and deriving novel relations by appropriately combining them. The system correctly inferred that two stimuli both opposite to a common stimulus are same as each other, a hallmark of flexible relational reasoning. Achieving this required NARS to represent not just “X relates to Y” but “X relates to Y in context Z” (where Z is SAME or OPPOSITE), and to have inference rules that respect these contexts and their combinatorial properties.

In sum, the AARR study demonstrated that NARS/ONA is capable of derived relational reasoning consistent with fundamental RFT principles. It exhibited mutual entailment (symmetric inference) and combinatorial entailment (transitive/combined inference) in both SAME and OPPOSITE contexts (Johansson, 2025; Johansson et al., 2025). This validates that symbolic relational operant behavior—the foundation of human language and cognition according to RFT—can be captured by a cognitive architecture when augmented with appropriate mechanisms for representing and reasoning about acquired relations.

Importantly, all of these relations were learned by NARS; the system did not innately “know” what Same or Opposite mean initially—it was trained via feedback in simulated MTS trials to establish those relational operants, just as a human might learn what “opposite” means through examples. This underscores the Machine Psychology principle that even highly abstract cognitive skills can be incrementally trained into an AGI system, rather than having to be pre-programmed.

## 10 Mapping NARS mechanisms to psychological processes

A key contribution of Machine Psychology is elucidating how specific computational mechanisms in NARS correspond to known psychological learning processes. Here, “mechanisms” refers to both built-in features of the NARS architecture (such as temporal inference, priority-based control, and variable introduction) and additional mechanisms introduced for the Machine Psychology research program (such as functional equivalence detection and acquired relations, as described in Sections 8, 9). By analyzing the studies above, we can draw direct parallels between NARS's operations and cognitive-behavioral functions:

1. Temporal and procedural inference → operant conditioning: NARS's ability to handle temporal sequences and form procedural implications (i.e., (context,action) → outcome relations) is what enables operant learning. In NARS, a procedural implication is a rule like “if X is observed and operation O executed, then outcome Y will be observed.” This mirrors the psychological concept of an operant contingency (Sd : R → Sr). NARS updates the truth value of such an implication based on success or failure, which has some resemblance to reinforcement strengthening or weakening a functional relation (Johansson, 2024b). Thus, NARS's temporal inductive rule (detecting that one event regularly follows another) serves as a model of how an organism might come to anticipate consequences (Wang, 2022). The operant experiments showed NARS adjusting its procedural knowledge just as a rat adjusts its lever-press strategy—in both cases, the underlying mechanism is the strengthening of an action–outcome expectation through repeated reinforced experience.
2. Priority-based control → attention and adaptive motivation: NARS's built-in resource management, particularly its priority-based task handling, maps onto the concept of attention in cognitive psychology. When NARS gives higher priority to a task that has recently yielded reinforcement (and perhaps demotes tasks that don't lead anywhere), it resembles the phenomenon of selection and focus on reinforcing stimuli that animals exhibit (e.g., a pigeon pecking the key that produces food and ignoring one that doesn't). This mechanism prevented NARS from wasting time on uneffective actions once it found a better strategy with a higher success rate, analogous to how animals allocate behavior toward more reinforcing options. In the reversal learning task, NARS's shifting of priority from the formerly correct response to the new correct response corresponds to extinction and re-acquisition: the system “de-emphasized” the old pattern of responding when it stopped paying off and focused on exploring alternatives until a new successful behavior was found (Johansson, 2024b). Psychologically, this is akin to attention shifting to a novel stimulus when an expected reinforcing consequence is omitted—a basic element of adaptive behavior.
3. Variable introduction → abstraction and generalization: the introduction of variable terms in NARS (e.g., using #1 as a placeholder in an implication) provides a direct analog to the cognitive ability to form categories or concepts that apply across instances (Wang, 2013). In humans, generalization often arises from recognizing commonalities and ignoring specifics; similarly, NARS uses variables to represent “any object” in a certain role. In the generalized identity matching study, variable introduction allowed NARS to formulate the rule “select the option that matches the sample” without being tied to a particular color or shape (Johansson et al., 2023). This mechanism models how humans derive abstract rules—by effectively substituting concrete details with variable terms. It is related to what developmental psychologists call schematization (forming a general schema from examples). Variable-based abstraction in NARS is also comparable to the role of generalization in classical conditioning (responding to a range of similar stimuli), though here it is more sophisticated: NARS was not just generalizing a response to similar stimuli, it was generalizing a relation (sameness) to entirely new stimuli. That aligns with how concept learning is understood in cognitive psychology, where one learns an idea like “sameness” that can apply to any content (Skinner, 1953; Hayes et al., 2001).
4. Derived implication and equivalence → stimulus equivalence and symmetry: to achieve functional and stimulus equivalence, NARS utilized its capacity to chain implications and generate new ones. When NARS observed A → outcome and B → same outcome, it could derive an implication between A and B (perhaps not with absolute certainty, but as a hypothesis). This models the psychological stimulus-stimulus relation that forms when two stimuli predict the same event (as in mediated conditioning). The mechanism introduced as “functional equivalence” (two-way implications) in NARS essentially binds together representations that have identical consequences (Johansson et al., 2024). Psychologically, this corresponds to forming an equivalence class—a process Sidman (2000) described in terms of reinforcement contingency overlap. Furthermore, NARS explicitly representing an equivalence (through symmetric implications like A⇒B and B⇒A) captures mutual entailment (symmetry), one of the core properties of relational responding. Humans demonstrate symmetry when, after learning A → B, they spontaneously infer B → A; NARS demonstrated exactly that by utilizing its inference rule for the inversion of relations under certain conditions (in NAL, if a relation is marked as symmetric or if a pair of implications can be combined, symmetry emerges). NARS's successful derivation of B1–C1 from A1–B1 and A1–C1 resembles transitivity (A = B, A = C yields B = C), which is another emergent property. These logical derivations in NARS align perfectly with the behavioral description of stimulus equivalence formation (Sidman, 2000; Hayes et al., 2001). In essence, NARS's acquired relations mechanism (Johansson, 2025) stands in for the operant learning of relational frames in humans—it takes specific trained relations and generalizes the network (yielding symmetry and transitivity) without further feedback, similarly as humans do after minimal training.
5. Contextual frames (implication with context) → relational context and conditional discrimination: NARS's extension to handle SAME and OPPOSITE as contextual qualifiers maps onto the idea of conditional discrimination and contextual control in psychology. In complex human learning, a cue can signal how to relate stimuli (e.g., “In this game, treat these as opposites”). NARS realized this by incorporating the context into the representation of a statement, effectively making relations three-term: (X × relation × Y). This is analogous to adding a contextual stimulus in a conditional discrimination that tells the subject which comparison is correct. The architecture needed to manage potentially conflicting relations (X same Y vs. X opposite Y in different contexts)—reflecting how humans keep track of multiple relational frames without confusion, thanks to context (Hayes et al., 2001). By successfully applying different inference rules depending on the relational context (e.g., if context = SAME then transfer properties directly; if context = OPPOSITE then transfer inversely), NARS replicated the conditional aspect of relational responding. This is related to the psychological concept of relative frames where, for instance, the presence of an “opposite” cue triggers one to derive the opposite relation rather than the same (Hayes et al., 2001; Roche et al., 2000). Essentially, NARS's context-tagged reasoning is performing the role of a conditional cue in human experiments, and the successful outcomes indicate that it functioned comparably, thereby mapping a high-level cognitive flexibility (switching relational rules) to a concrete system of tagged logical rules.
6. Implication and term logic → analogical reasoning: although not explicitly highlighted in the experiments above, it's worth noting that NARS's general reasoning ability also gives it a form of analogy-making. For example, in AARR tasks, seeing the pattern A1 → B1 (same) and A1 → C1 (same) and by analogy in the second network A2 → B2, A2 → C2, NARS can analogically complete patterns (Johansson, 2019b). This capacity comes from NARS's ability to instantiate variables and follow isomorphic structures in its relational network—akin to analogical mapping in humans (Gentner's structure mapping theory). Therefore, the same NARS machinery responsible for combinatorial entailment also underlies analogical reasoning (Wang, 2013), mapping one set of relations onto another if they share a structural resemblance. This was implicitly in play when NARS applied what it learned in one relational network (A1–B1–C1) to a parallel one (A2–B2–C2) in the experiments.

In conclusion, every major component we added to NARS corresponds to a well-known psychological mechanism:

- Temporal credit assignment in NARS ↔ reinforcement learning (reinforcement schedules).
- Variable-based generalization ↔ concept formation and general rule learning.
- Symmetric and transitive inference ↔ emergent stimulus equivalences.
- Contextual modulation ↔ conditional discrimination and rule-governance.

Table 1 provides a summary of how the four empirical studies reviewed in this article map psychological processes to specific NARS mechanisms and the corresponding layers of Non-Axiomatic Logic (Wang, 2013).

**Table 1:** Overview of psychological processes, NARS mechanisms, NAL layers (Wang, 2013), and references for the four Machine Psychology studies.

**Psychological process**	**NARS mechanisms**	**NAL layers**	**References**
Operant conditioning	Temporal reasoning and procedural reasoning	7–8	Johansson, 2024b
Generalized identity matching	+Abstraction	+6	Johansson et al., 2023
Functional equivalence	+Implications	+5	Johansson et al., 2024
Arbitrarily applicable relational responding	+Acquired relations	+4	Johansson, 2025; Johansson et al., 2025

This alignment not only validates NARS as a model of those psychological phenomena, but it also gives us insights into the computational nature of cognition. It suggests that intelligence might be achieved by an architecture that integrates these mechanisms—learning from feedback, forming abstract generalizations, relating concepts flexibly, and using context to control behavior (Johansson, 2024a). The Machine Psychology approach therefore offers a blueprint: by systematically adding mechanisms that mirror psychological learning processes (operant relations, generalization, equivalence relations, relational framing), we can inch an AI system closer to human-like cognitive capabilities.

## 11 Machine Psychology as a developmental roadmap to AGI

The progression from operant conditioning to arbitrarily applicable relational responding in NARS illustrates a stepwise developmental roadmap for building up a mind, much like the developmental trajectory of human cognition. In human development, infants first acquire sensorimotor skills and basic operant behaviors (crying leads to being fed, pressing a button yields a toy's sound), then they learn to recognize and generalize simple concepts (identifying objects, categories), later they understand equivalence and naming relations (a word stands for an object), and eventually they master complex symbolic reasoning and relational thinking (language, analogies, theory of mind) (Piaget, 1954; Hayes et al., 2001). Machine Psychology intentionally mirrors this sequence in an artificial system (Johansson, 2020, 2024b). By doing so, it provides a structured pathway for AGI development, with each stage building on the foundations of the previous.

One advantage of this staged approach is that it ensures each cognitive ability is functionally validated before moving to the next. In our case, we confirmed NARS could adaptively learn from consequences (a necessary condition for autonomous agents) before expecting it to generalize concepts. We then made sure it could generalize concepts before expecting it to handle equivalences and then contextual relations. This reduces the complexity at each step and allows us to identify which mechanism is responsible if something fails. It's akin to how educational curricula build from simple to complex topics, ensuring prerequisites are met. In contrast, many end-to-end AI approaches (for example, attempting to train a very deep network on a complex task directly) can be brittle or opaque in what they learn. The Machine Psychology roadmap, by being grounded in well-understood behavioral principles, provides explainability and diagnosability at each stage (Johansson, 2024a). For instance, if NARS had struggled with functional equivalence, we would pinpoint that the mechanism for combining implications needed adjustment, rather than guessing among many potential causes.

Another benefit is that this progression integrates learning and reasoning in a cumulative way. Early operant learning gave NARS an experiential base (much like a child learning basic cause-effect), which likely made later learning more efficient because NARS could reuse its procedural knowledge structure when learning more abstract tasks (Johansson, 2024b). When NARS tackled relational reasoning, it leveraged both its logical inference and its learned knowledge (like how to approach a matching task, how to interpret a reinforcement signal, etc.). This synergy between accumulated knowledge and new reasoning challenges is reminiscent of human cognitive development: each stage doesn't start from scratch, but refines and expands prior capabilities. The result is a coherent system where low-level and high-level cognition are not separate modules but continuous aspects of the same architecture. In AGI terms, this addresses the long-standing issue of integrating symbolic and sub-symbolic abilities—here, the sub-symbolic (learning from raw feedback) and symbolic (manipulating abstract relations) coexist in NARS as part of one framework.

This developmental approach also naturally leads to increasingly general intelligence. Initially, NARS's intelligence was narrow (solving a particular discrimination task), but by the final stage, it was demonstrating very general reasoning patterns (applying the idea of “opposite” to arbitrary new situations, for example). Achieving AARR suggests a system with the rudiments of language and logic, since arbitrary symbols can control its behavior and it can derive implicit relations—a cornerstone of general problem-solving and communication. Indeed, Hayes et al. (2001) argue that once a organism (or machine) masters AARR, it possesses the core of human language ability, and thus can acquire all manner of knowledge that is transmissible via language. We can see a future Machine Psychology step where the system is taught linguistic frames like “bigger than,” “before/after,” “perspective of self/other” (deictic frames related to theory of mind). Each of these would further elevate the system's cognitive repertoire. Notably, Johansson (2020) suggests that a full roadmap to human-level AGI would involve training an AI on progressively complex relational frames, from simple non-arbitrary relations to very high-order abstractions. The work reviewed here accomplished the early and mid stages of that ladder (identity, equivalence, opposition). One can imagine next adding hierarchical relations (e.g., category membership frames) or temporal relations (before/after, enabling understanding of sequences), and deictic relations (“I–You,” “Here–There,” which underpin theory of mind and perspective taking).

By following such a curriculum, an AGI could potentially develop a theory of mind—the ability to understand others' perspectives and intentions—as a natural extension. For example, “I” vs. “you” frames are arbitrarily applicable relational frames that children learn; training NARS on these could allow it to simulate reasoning about what another agent knows or wants (as it would have internal relational structures to represent different viewpoints). Early evidence that this is plausible comes from NARS's handling of context: it already can apply different rules in different contexts (SAME vs. OPPOSITE). Extending that, a context could be “from my perspective” vs. “from your perspective,” and NARS could be trained to derive that something I know, you might not know (a sort of opposite in knowledge frame). This is speculative (but see Wang et al., 2018), but it illustrates how Machine Psychology provides a tangible route to incorporate Theory of Mind into an AI: treat it as just another relational frame to be operantly learned and practiced.

The developmental trajectory showcased by Machine Psychology also invites measurement-level analogies between NARS mechanisms and neural substrates, though it is important to note that these are structural parallels rather than causal claims. For example, NARS's temporal credit assignment for action–outcome contingencies is analogous, at a descriptive level, to the role of basal ganglia and dopaminergic circuits in reinforcement-based learning; NARS's priority-based attentional control shares functional similarities with prefrontal cortex modulation of task-relevant processing (Lofthouse, 2019); and NARS's variable binding and relational reasoning parallel the role of frontoparietal networks in working memory and relational integration. However, from a contextual behavioral science perspective, the explanatory variable remains the functional relationship between behavior and environment—the contingencies of reinforcement and relational learning—rather than any particular neural implementation. Neural correlates represent where these behavioral processes are manifested in biological organisms, not why they work. NARS is best understood as a computational model of these functional relationships, and any neural analogies serve primarily as heuristic bridges between AI modeling and neuroscience research, not as mechanistic explanations of NARS's own operation.

Finally, Machine Psychology as a roadmap emphasizes empirical rigor and incremental progress in AGI. It addresses the critique that AGI research has been too aspirational without clear measurable milestones (Thórisson et al., 2024; Wang, 2012). Here, each study acts as a milestone—e.g., “AI system passes generalized identity matching test,” “AI system demonstrates stimulus equivalence and transfer of function,” etc. These are objective behavioral criteria, analogous to developmental milestones in children (like passing the mirror self-recognition test or false-belief test in humans). Such milestones allow the community to track progress in AGI in a more standardized way, rather than relying on subjective impressions or one-off demonstrations. This also encourages reproducibility—other researchers can attempt the same tasks with different architectures, and either achieve them (thus validating the approach) or fail (thus highlighting what might be uniquely powerful about NARS or what's missing in other systems).

In summary, Machine Psychology offers a systematic path toward AGI, grounded in the idea that to build a mind, one should follow the way minds naturally develop—layer by layer, skill by skill. The results so far support the feasibility of this path: by the time NARS handled AARR, it was operating with a flexibility and generality far beyond where it started. While there is much further to go (language fluency, learning from instruction, creative problem solving, etc.), the journey outlined—operant learning → simple relations → complex relations—provides a map for reaching those advanced abilities.

## 12 Limitations and future directions

Several limitations of the current work should be noted. First, all experiments were conducted in controlled environments with small stimulus sets (2–6 stimuli per task) and discrete, pre-encoded representations. It remains to be seen how NARS/ONA scales to richer environments with larger stimulus spaces and continuous sensory input (Wang, 2013; Hammer et al., 2023b). Second, all studies relied on explicit reinforcing consequences; incorporating intrinsic motivation mechanisms would be important for more autonomous developmental progression. Third, it is worth distinguishing two challenges: *brittleness* (failure to generalize beyond training), which Machine Psychology directly addresses through its progressive generalization studies, and *opacity* (difficulty understanding system outputs), which NARS's symbolic architecture mitigates through inspectable rules and truth values, though its non-deterministic processing can complicate exact behavioral prediction. Finally, the results reported here depend on both the NARS *architecture* (Non-Axiomatic Logic, experience-grounded semantics, resource-bounded reasoning) and specific *additional mechanisms* introduced in OpenNARS for Applications for this research program (functional equivalence detection, acquired relations). Whether other NARS implementations or architectures with similar design principles would yield comparable results remains an empirical question.

Future directions include extending the range of relational frames to hierarchical, temporal, and deictic relations (I/You, Here/There)—the latter being critical for theory of mind (Johansson, 2020)—as well as multi-agent tasks and the integration of linguistic input to test rule-governed alongside contingency-shaped behavior (Hayes et al., 2021).

## 13 Conclusion

Machine Psychology provides a principled, developmentally grounded framework for advancing Artificial General Intelligence by anchoring AI progress in well-established learning psychology. Using the Non-Axiomatic Reasoning System (NARS) as a unifying cognitive architecture, the studies reviewed here demonstrate a cumulative progression from operant learning to generalized abstraction, functional equivalence, and arbitrarily applicable relational responding. Crucially, these competencies were acquired within a single adaptive system operating under conditions of uncertainty and limited resources, mirroring key constraints of biological cognition. This shows that increasingly abstract and symbolic forms of reasoning can emerge through incremental learning, rather than requiring task-specific architectures or pre-programmed symbolic knowledge.

At the same time, the results highlight important challenges for future work. Scaling these mechanisms to richer environments, supporting more autonomous exploration, and integrating perceptual and linguistic interfaces remain open problems. Nevertheless, the Machine Psychology approach offers a clear empirical roadmap: by treating cognitive abilities as learnable repertoires validated through behavioral benchmarks, it enables systematic, interpretable, and cumulative progress toward general intelligence. Grounded in psychological theory and realized in a functioning reasoning system, this framework provides both a blueprint for AGI development and a testable model of how complex cognition can be constructed from simpler learning processes.

## In memoriam

This paper is dedicated to Tony Lofthouse, our long-time friend, colleague, and collaborator, who sadly passed away on October 25th, 2025.
